# Atomic Simulation of the Coalescence and Melting Process of Ag_309_ and Cu_309_ Clusters

**DOI:** 10.3390/nano16181194

**Published:** 2026-09-21

**Authors:** Haiyong Shen, Jinhan Liu, Lin Zhang

**Affiliations:** 1 School of Design and Art, Shenyang Jianzhu University, Shenyang 110168, China; 2 Key Laboratory for Anisotropy and Texture of Materials (Ministry of Education), Northeastern University, Shenyang 110819, China; 3 Department of Materials Physics and Chemistry, School of Materials Science and Engineering, Northeastern University, Shenyang 110819, China

**Keywords:** Ag, Cu, coalescence, molecular dynamics, heating

## Abstract

The coalescence of Cu_309_ and Ag_309_ clusters and the subsequent thermal evolution of the resulting Cu-Ag alloy clusters are systematically investigated via molecular dynamics simulations. The effects of initial coalescence distance and contact orientation on the potential energy, free energy, shape factor, and atomic packing structures are examined in detail. The results demonstrate that the activation energy for coalescence is influenced by both initial coalescence distance and the contact orientation, with the facet-to-facet orientation generally exhibiting the highest energy barrier. During the initial coalescence stage, the free energy remains essentially constant until the clusters come into contact, after which distinct evolutionary pathways emerge depending on the orientation. Upon heating, the average potential energy reveals multiple stages of atomic rearrangement, structural transition, and melting, with the transition temperatures varying significantly with the initial conditions. Shape factor analysis indicates that most clusters evolve toward a nearly spherical morphology at high temperatures, while atomic packing visualizations confirm the formation of core@partial-shell, incomplete icosahedral, and fully molten configurations depending on the temperature and initial parameters. This work provides atomic-scale insights into the coalescence behavior and thermal stability of Cu-Ag alloy clusters, offering theoretical guidance for the design and synthesis of bimetallic nanoclusters with tailored structures.

## 1. Introduction

Metal nanoclusters exhibit significant potential for applications in fields such as nanodevices, molecular imaging, biosensors, and biomedicine due to their unique properties, including excellent photostability, high luminescence efficiency, low toxicity, good water solubility, and straightforward synthesis methods [1,2,3,4,5,6]. Furthermore, owing to their sub-nanometer size, metal nanoclusters demonstrate quantum confinement effects and discrete energy level structures, which endow them with distinctive catalytic and fluorescent characteristics [7,8,9,10]. Among these, silver nanoparticles have become the most widely used noble metal due to their high electrical and thermal conductivity, excellent antibacterial properties, and relatively low cost [8,11]. In recent years, silver nanoclusters have also attracted extensive attention for their superior fluorescence properties and biocompatibility, making them more readily obtainable compared to other metal nanoclusters [12]. This has provided a significant foundation for their applications in various fields such as catalysis, photo-absorption, the biomedical field, etc. [13]. Recently, studies on replacing silver atoms in silver nanoclusters with copper have garnered considerable interest. This is primarily because copper is less expensive and exhibits higher electrical conductivity than silver, while copper nanoparticles themselves possess favorable electrical, optical, and catalytic properties [14,15,16]. As a result, Cu-Ag nanoalloys have emerged as a research hotspot in the field of nanomaterials.

Bimetallic clusters have garnered extensive attention in recent years due to their unusual properties that have not been observed in monometallic clusters [17]. These clusters are also deemed to hold diverse application prospects, with remarkable performance particularly in the fields of catalysis, optics and magnetism [18,19,20]. Furthermore, depending on the specific selection of the two metals and their bulk miscibility characteristics, bimetallic clusters can form a variety of mixed configurations, including core–shell structure, sub-cluster segregation structure, mixed structure and three-shell structure [21]. Cu-Ag bulk is a typical eutectic alloy. In the form of nanoalloys, it can be widely applied in fields such as catalysts, sensors, electrodes, and lead-free solders [22,23,24]. At the nanoscale, binary alloys are more miscible than their corresponding bulk counterparts and can form various structures. For instance, Ferrando et al. systematically investigated the structural stability of Cu-Ag nanoalloys with a total atomic number ranging from 34 to 45 based on density functional theory (DFT) calculations. The results demonstrated that all Cu-Ag nanoalloys within this size range exhibit the pseudo-icosahedral (pIh) structure [25]. Núñez et al. combined genetic algorithm-based global optimization with the Gupta semi-empirical many-body potential to study the structure and chemical order of Cu-Ag nanoalloys containing 34, 38, and 98 atoms, and found that their lowest-energy structures are all Cu-core/Ag-shell configurations [26]. Molayem et al. determined the global energy minimum structures of the Cu_m_Ag_n_ (m + n = 2~60) nanoalloys, and the obtained configurations mainly include icosahedral (Ih) structure, pseudo-icosahedral (pIh) structure, and pentagonal symmetric structure [27]. Roncaglia et al. reported that the global energy minimum structure of the Cu_55_Ag_45_ nanoalloy is a 55-atom Mackay icosahedron covered by an incomplete anti-Mackay shell [28]. Laasonen et al. performed computational simulations on Cu-Ag nanoalloys with atomic numbers up to 1400, and the results indicate that Cu-Ag nanoalloys tend to form core–shell structures, where the core can either be located at the central position or be off-center [29].

A key method for nanoalloy synthesis relies on the collision and coalescence of nanoclusters. Due to its dependence on surface area, the coalescence process is of great importance in catalysis and corrosion, leading to widespread interest in recent years [30]. Although experimental observations of the coalescence process remain limited at present, numerous simulation studies have been conducted to explore the possibility of this process. In studies of cluster coalescence, the primary focus has been on metallic nanoclusters such as Cu, Ag, Al, and Au [31,32,33]. By selecting clusters with distinct physical properties and controlling the coalescence time and temperature during the merging of different metal types, it is possible to synthesize alloy clusters with specific structures and morphologies. For instance, Mizuno et al. obtained bimetallic nanoclusters with core–shell, mixed, and hetero-structured configurations through the coalescence of different metal clusters [34]. Furthermore, studies have revealed that bimetallic nanoclusters with varying structures and chemical compositions exhibit markedly different structures from the collision complexes under different collision conditions [35]. Additionally, the coalescence of nanoparticles, such as Ag and Cu nanoparticles, has emerged as a new option in the field of high-temperature device packaging. Tian et al. employed the Monte Carlo method to characterize the distinct stages involved in the coalescence of Ag and Cu clusters [36]. The process initiates with the mutual diffusion and contact of adjacent Ag and Cu clusters, leading to the formation of a neck region between them. Subsequently, the coalesced cluster undergoes structural deformation and eventually reaches a new equilibrated state. Grammatikopoulos et al. used molecular dynamics to rationalize the formation of metastable core–shell Cu-Ag nanoalloys during coalescence and highlighted the difference in coalescence mechanisms between Ag-rich and Cu-rich nanoparticles [37]. They further proposed controlled nanoparticle coalescence as a novel strategy for designing Cu-Ag nanoparticles with unique core@shell structures. Separately, Liang et al. investigated the effects of atomic ratio and interface structure on Cu-Ag nanoalloys during coalescence via molecular dynamics simulations. Their results indicate that a higher Ag content promotes the formation of a core–shell structure. Different atomic and interfacial arrangements were found to lead to distinct coalescence behaviors in the initial contact stage [38]. Currently, the lack of a detailed understanding of the coalescence process between Ag and Cu clusters at the atomic scale significantly hinders the structural design and preparation of Cu-Ag alloy clusters. Furthermore, whether different initial coalescence conditions can alter the microstructure and properties of the resulting Cu-Ag alloy clusters remains a question worthy of in-depth investigation.

In the present work, atomic simulations using molecular dynamics were performed to explore the coalescence processes between two Cu_309_ and Ag_309_ clusters as well as the structural changes in the coalesced binary clusters at elevated temperatures. Here, the number 309 is one typical magic number in the clusters, and they present perfect icosahedral (Ih) geometry [39]. The thermal behaviors of these clusters were demonstrated by potential energy per atom and shape factors, as well as visual atomic packing within the framework of the embedded atomic method (EAM).

## 2. Model and Simulation

In this paper, the interaction among the atoms is described by the EAM form, which was proposed by Williams [40]. The total potential energy of the system $E_{tot}$ is determined by

(1) $$E_{tot}=\frac{1}{2}\sum_{ij}V_{ij}\left(r_{ij}\right)+\sum_{i}F_{i}\left({\bar{\rho }}_{i}\right),$$

(2) $${\bar{\rho }}_{i}=\sum_{j\neq i}\rho_{j}\left(r_{ij}\right),$$

where $V_{ij}(r_{ij})$ is the potential energy between atoms *i* and *j* having a distance of *r_ij_*_,_ and $F_{i}(\bar{\rho_{i}})$ is the embedding energy with an electron density of $\bar{\rho_{i}}$ at the position of the atom *i*. The density value is obtained from the superposition and sum of the electron density from the nearest neighboring atoms of the atom *i*. $\rho_{i}$ is the electron density of the atom *j*.

**Figure 1:**
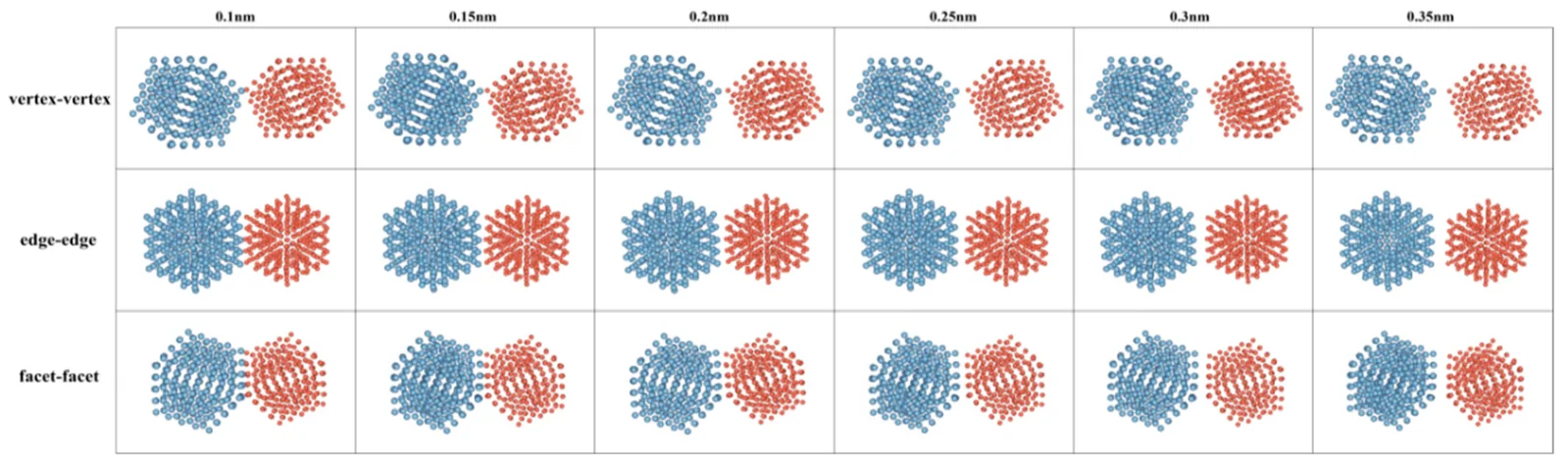
Initial packing structures of the clusters before coalescence.

The simulations were carried out in the NVT ensemble using an Andersen thermostat. By solving Newton’s equations, we could obtain the positions and velocities of each atom, and a predictor-corrector algorithm was used to integrate the equations of motion. Throughout the simulations, a time step of 1.6 × 10^−15^ s was used. At each temperature, the system was first fully equilibrated in 1.592 ns before running to accumulate statistics, and the atomic trajectories and the energy can be obtained by calculating the average of the energy from the subsequent 8 × 10^−3^ ns, corresponding to the last 5000 time steps of the whole 1,000,000 time steps in the simulations. The time steps used for statistics should be larger than 3N-6 in the simulated system, where N is the number of atoms in the system. The increase in the statistical time will lead to the distortion of the shape of the calculated particle from average coordinates owing to the rotation of the cluster. Therefore, at each temperature, we determine the packing configuration with the lowest energy in the last 5000 time steps. Initially, we constructed an MD simulation cell with 20a_0_ × 20a_0_ × 20a_0_ (the Ag lattice constant a_0_ is 4.09 Å), and the simulated clusters are put in the center of the cell. Here, the box size of the simulated central cell is large enough to avoid the interaction of the atoms in this central cell with the other atoms in its 26 neighbor imaging cells under periodic boundary conditions, and this size remains constant at simulated temperatures. The Ag_309_ and Cu_309_ clusters have an Ih configuration after structural relaxation at 300 K. Subsequently, the obtained different clusters were merged to obtain the initial structure shown in Figure 1, in which the Ag atoms are marked in blue, and the Cu atoms are marked in orange. We have chosen different coalescence distances (0.1, 0.15, 0.2, 0.25, 0.3, 0.35 nm), marked as *D_c_*. Also, we simulated the coalescence process of clusters that are coalesced in different orientations, including vertex-to-vertex, edge-to-edge and facet-to-facet, as shown in Figure 1. The initial clusters from Figure 1 were continuously heated from 300 K to 1300 K at an increment of 50 K, that is, 300 K→350 K, 350 K→400 K, etc. On heating, the structure of the last step, which was obtained from the structural relaxation at each temperature, was simulated as the initial structure at the next temperature.

The moment of inertia can reflect atomic positions and mass distribution. As a tensor, it can be determined by:

(3) $$I_{ij}=\sum_{iatom=1}^{N}m_{Cu/Ag}^{i}\times \left(x_{iatom}^{i}-x_{c}\right)\times \left(x_{iatom}^{j}-x_{c}\right)\;(i\;\mathrm{or}\;j=1,2,3),$$

(4) $$x_{c}=\sum_{iatom=1}^{N}m_{Cu/Ag}^{i}\times x_{iatom}^{i}/\sum_{iatom=1}^{N}m_{Cu/Ag}^{i}\;(i=1,2,3),$$

where *m_Cu_*_/*Ag*_ is the mass of the Cu atom or the Ag atom, $x_{c}$ is the center of mass, and $x^{i}$ or $x^{j}$ is the coordinate of the *iatom*. *i* (or *j*) is 1, 2, 3, corresponding to the *x*, *y* and *z* axes, respectively. Three principal values of *I*^1^, *I*^2^ and *I*^3^ are obtained by diagonalization of the moment of inertia, where *I*^1^ and *I*^3^ are the maximum and minimum values. The shape factor can be defined as:

(5) $$F_{shape}=I^{1}/I^{3},$$

If the shape factor value is closer to 1, the cluster has a nearly spherical shape.

The free energy consists of translational and rotational parts after ignoring the contributions from electrons and vibrations. At a given temperature, the change in free energy from rotation becomes apparent, while the contribution of translational motion is expressed as a constant value. At temperature T, the translational free energy can be given by −*k_B_T* × *lnZ_t_*, where *k_B_* is the Boltzmann constant and the *Z_t_* translation distribution function is determined by temperature. The free energy generated by rotation is given by −*k_B_T* × *lnZ_r_*, where the contribution of the *Z_r_* rotational distribution function comes from the spatial distribution of the component atoms, related to the product of the three principal values of the moment of inertia, temperature and molecular symmetry in these nanoparticles.

## 3. Results and Discussion

**Figure 2:**
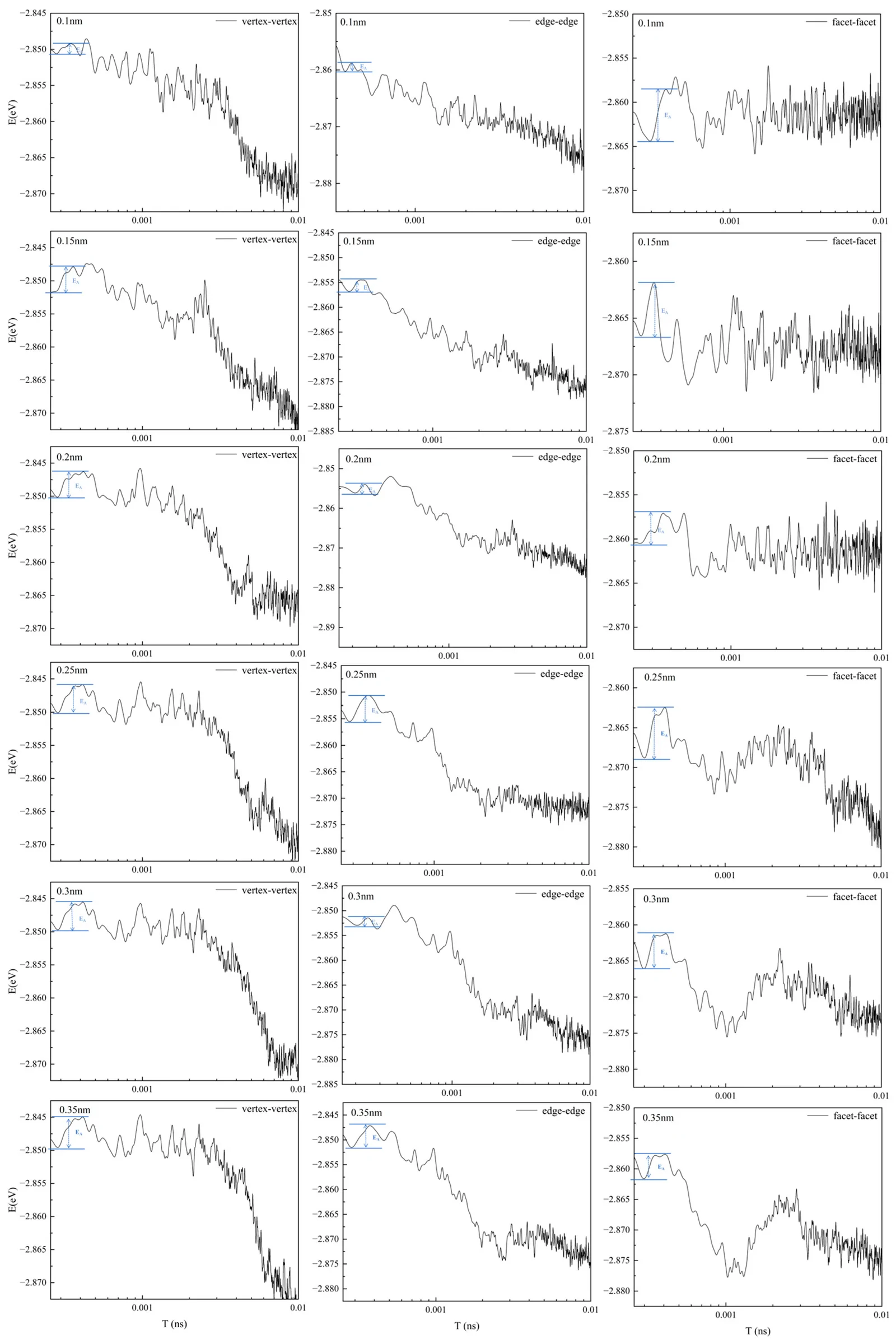
Potential energy varying with the structural relaxation time at 300 K.

Figure 2 shows the variation in the potential energy for Ag_309_ and Cu_309_ clusters with Ih packing structure at 300 K, where the time is plotted on a logarithmic scale. As shown in the figure, when the initial coalescence distance is 0.1 nm, the potential energy of the clusters coalescing in the vertex-to-vertex orientation first decreases from a high value to a local minimum of −2.851 eV within a very short time interval, and then increases to a local maximum of −2.849 eV. The potential energy difference associated with this process corresponds to the energy barrier that must be overcome during the mutual approach of the two clusters. As the simulation proceeds, the potential energy of the clusters shows a damped oscillatory behavior. Following several oscillations, the potential energy decreases with fluctuations over the time interval from 0.0048 to 0.0112 ns, suggesting that the coalescence of the two clusters is complete. As the simulation proceeds, the potential energy gradually decreases with fluctuations. For the clusters coalescing in a facet-to-facet configuration, the potential energy drops to −2.864 eV at 0.00029 ns and then rises to −2.859 eV at 0.00037 ns. Between 0.00037 and 0.0112 ns, the potential energy shows repeated oscillations. The energy gaps between local minima and maxima in the early coalescence stage are strongly dependent on the initial contact orientation and coalescence distance for Ag_309_ and Cu_309_ clusters. Table 1 presents the energy gaps between the local potential-energy minima and maxima in the early coalescence stage. These gaps are denoted as the “activation energy” $E_{A}$, which corresponds to the energy barrier that must be surmounted for the coalescence of the Ag and Cu clusters to initiate, signifying the maximum barrier to be overcome as the clusters evolve from isolated entities to the coalescence process.

As can be seen from Table 1, when the coalescence distance *D_c_* is 0.1, 0.15, 0.2, and 0.3 nm, the activation energy is largest for clusters coalescing in the facet-to-facet configuration, followed by those in the vertex-to-vertex configuration, and smallest for those in the edge-to-edge configuration. For clusters with an initial coalescence distance of 0.25 nm, the vertex-to-vertex configuration exhibits the smallest activation energy, while the facet-to-facet configuration shows the largest. When *D_c_* = 0.35 nm, the activation energies for the three coalescence configurations are relatively close to one another.

**Figure 3:**
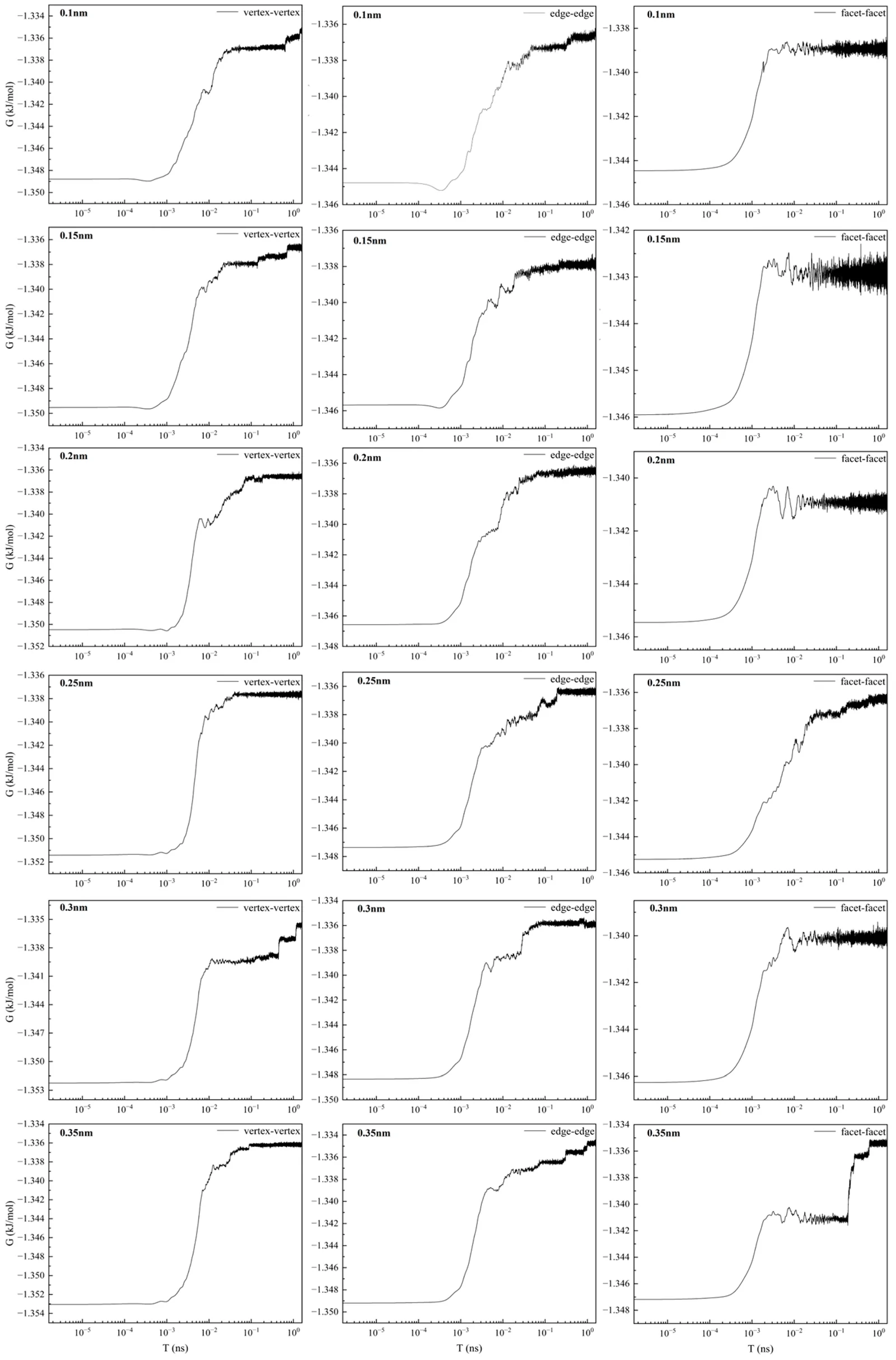
Free energy varying with time for the coalescing clusters with different orientations and distances at 300 K.

Figure 3 shows the variation in free energy with time for the clusters with different orientations and distances at the initial stage of coalescence at 300 K, where the time is given in logarithmic form. The free energy of the pure Ag_309_ cluster at 300 K is −1.2648 kJ/mol, while that of the Cu_309_ cluster at 300 K is −1.2554 kJ/mol. The variation in free energy shown in the figure primarily reflects the changes in packing structure and overall morphology of the Cu and Ag clusters during the coalescence process. In the time range from 1.6 × 10^−6^ ns to 1.34 × 10^−6^ ns, corresponding to the initial coalescence stage, the free energy of the clusters remains essentially constant, indicating that the Cu and Ag clusters have not yet come into contact during this stage. After that, the free energy changes in the coalescing clusters present different behaviors. When *D_c_* = 0.1 nm, the free energy of the vertex-to-vertex coalescing clusters shows an initial slight decrease followed by a sharp increase in the time range from 1.34 × 10^−6^ ns to 0.022 ns. At this stage, the clusters begin to attract one another, establish contact, and initiate the formation of a new cluster. With increasing simulation time, the free energy of the clusters exhibits small-amplitude oscillatory behavior, indicating that the coalescence of the clusters has been completed. For the clusters coalescing in the edge-to-edge orientation, the free energy fluctuates with an increasing trend over the time interval from 1.34 × 10^−6^ ns to 0.012 ns, during which the clusters begin to coalesce. From 0.29 ns to 0.44 ns, the coalescence is already complete. However, the free energy exhibits a notable increase, indicating a change in the overall shape of the cluster. For clusters coalescing in the facet-to-facet configuration, the coalescence completion time is shorter than that of the former two configurations. For clusters coalescing in the vertex-to-vertex and edge-to-edge configurations, the initial free energy decreases with increasing coalescence distance. In addition, at the same initial coalescence distance, the free energy of clusters coalescing in the vertex-to-vertex configuration is significantly lower than that of the other two configurations.

**Figure 4:**
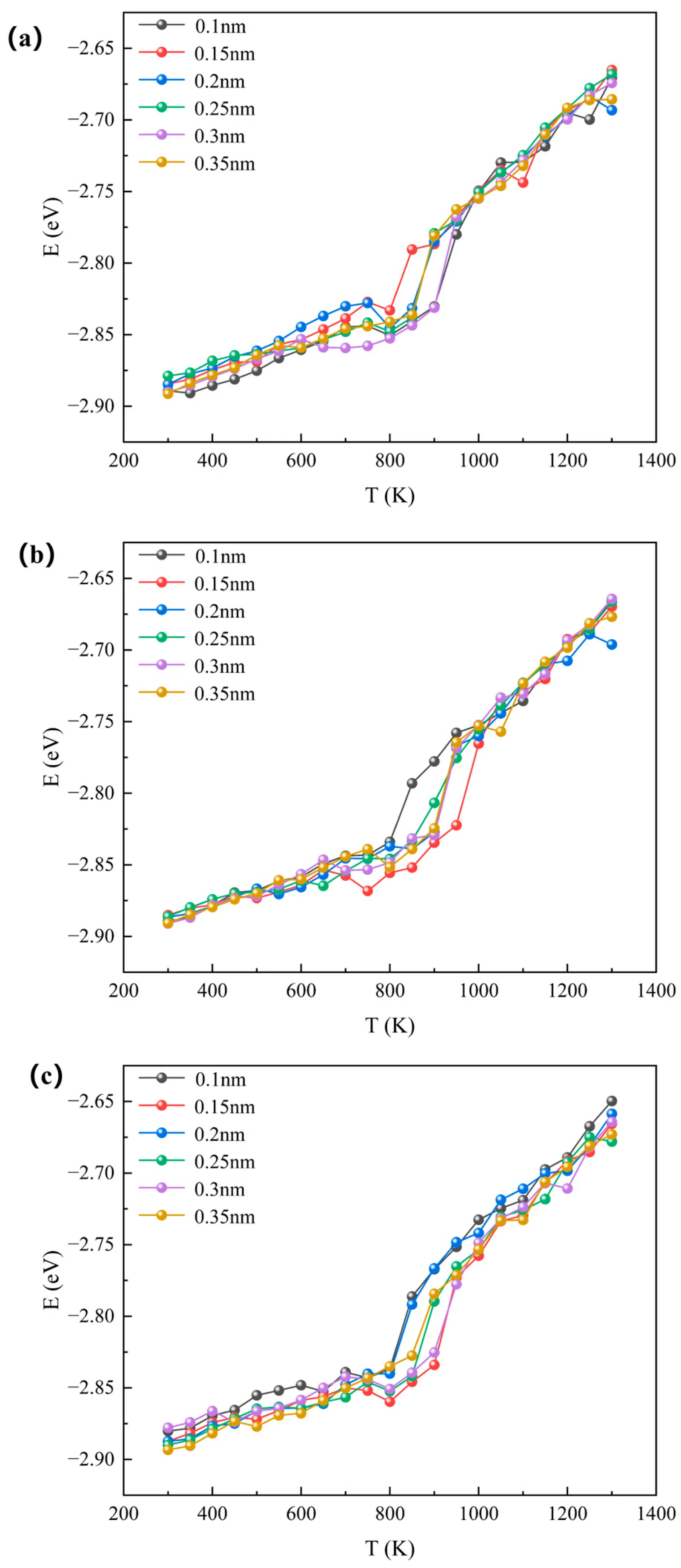
Variation in the atomic average potential energy of the coalesced clusters as a function of temperature. (**a**) vertex-vertex; (**b**) edge-edge; (**c**) facet-facet.

Figure 4 shows the variation in the average potential energy with temperature during the heating process for the Cu-Ag alloy clusters formed after the coalescence of Cu_309_ and Ag_309_ clusters under different initial coalescence distances and contact orientations. During the heating process, changes in atomic positions within the clusters induce variations in local structure and interatomic distances, which in turn alter the embedding energy and pairwise potential energy components of the total potential energy of the system. Consequently, the evolution of the average potential energy of the clusters can reflect the changes in the atomic packing structure. As can be seen from Figure 4a, for the Cu-Ag clusters coalesced in the vertex-to-vertex orientation, the average potential energy at 300 K is highest for those with an initial coalescence distance of 0.25 nm. In contrast, the average potential energies of the clusters with initial coalescence distances of 0.1, 0.3, and 0.35 nm are lower and relatively close to one another, indicating a higher degree of atomic ordering in these three clusters at this temperature. When *D_c_* = 0.1 nm, the potential energy of the Cu-Ag clusters exhibits a gradual upward trend in the temperature range from 350 K to 700 K, indicating that continuous atomic rearrangement occurs within the alloy clusters and that the atomic orderliness progressively decreases. As the temperature rises from 750 K to 800 K, the average potential energy of the alloy clusters shows a slight decrease, suggesting that the cluster system tends to evolve toward a more stable configuration with lower energy. With a further increase in temperature, the average potential energy of the Cu-Ag clusters exhibits a fluctuating upward trend, and a sharp surge is observed in the range of 900 K–950 K, indicating that a structural transition occurs within the clusters and that the degree of atomic disorder increases significantly. The variations in the average potential energy curves for the alloy clusters with initial coalescence distances of 0.15, 0.2, and 0.25 nm are similar to those with an initial coalescence distance of 0.1 nm. When *D_c_* = 0.3 nm, the average potential energy curve of the coalesced clusters exhibits a trend of first increasing, then slightly decreasing, and subsequently increasing again. For the alloy clusters with an initial coalescence distance of 0.35 nm, the average potential energy increases slowly over the temperature range from 300 to 850 K, without any notable abrupt change, indicating that although atomic thermal motion intensifies with increasing temperature during this process, no significant structural transition occurs. As can be seen from Figure 4b, the six Cu-Ag clusters coalesced in the edge-to-edge configuration exhibit relatively similar average potential energy values in the low-temperature region. As the temperature increases, the average potential energy of each alloy cluster generally shows a fluctuating upward trend. For the clusters coalesced in the facet-to-facet configuration (Figure 4c), at 300 K, the alloy clusters with initial coalescence distances of 0.1 nm and 0.3 nm have the highest average potential energies, whereas those with initial coalescence distances of 0.3 nm and 0.35 nm have the lowest.

**Figure 5:**
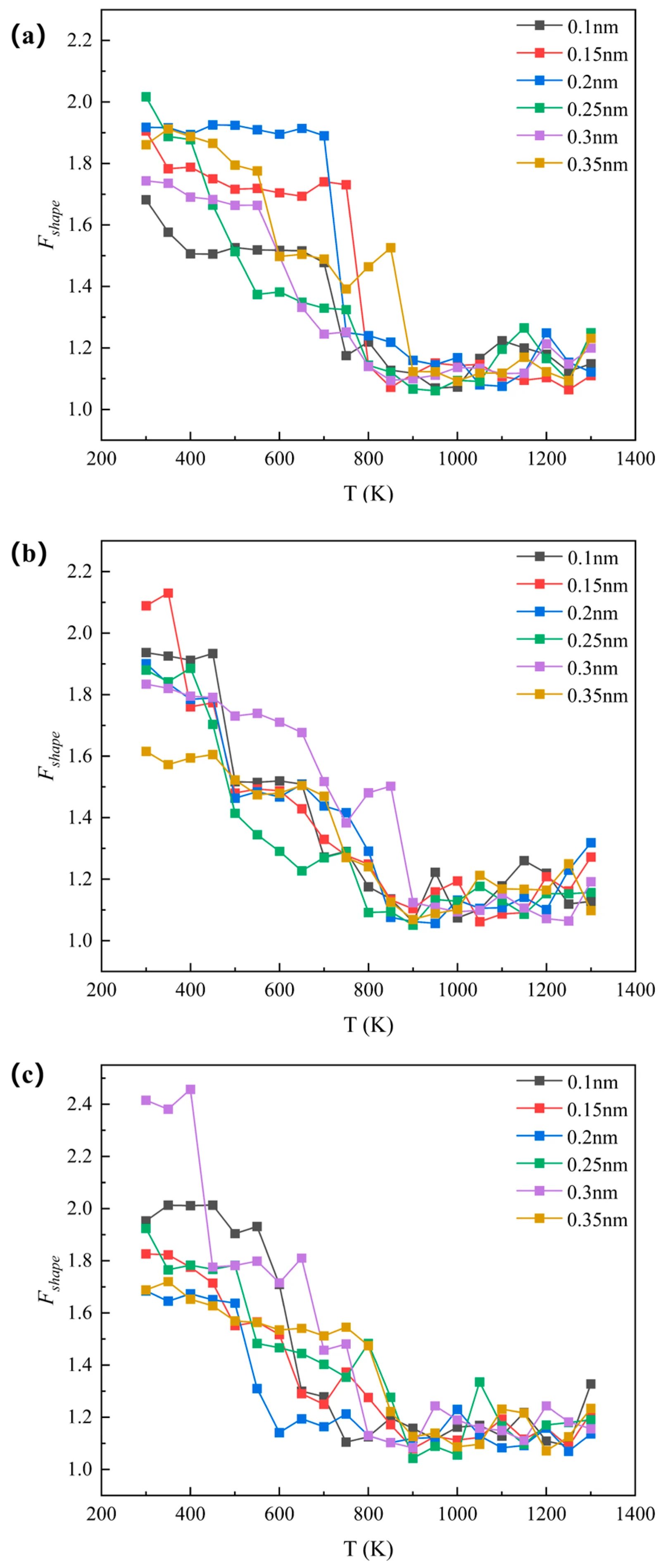
Variation in shape factor of the coalesced clusters as a function of temperature. (**a**) vertex-vertex; (**b**) edge-edge; (**c**) facet-facet.

Figure 5 shows the variation in the shape factor with temperature during the heating process for Cu-Ag clusters formed by coalescence at different initial coalescence distances and contact orientations. In the figure, a shape factor value close to unity indicates that the overall shape of the cluster is nearly spherical. As can be seen from Figure 5a, for the Cu-Ag clusters coalesced in the vertex-to-vertex orientation at 300 K, the shape factor is lowest for those with an initial coalescence distance of 0.1 nm. In the temperature range of 300 K–400 K, the shape factor of this cluster shows a decreasing trend, indicating that the degree of coalescence of the Cu-Ag clusters gradually improves. When the temperature rises from 700 K to 750 K, the shape factor of the coalesced cluster decreases substantially, suggesting that the overall cluster shape becomes more spherical. For the coalesced clusters with an initial coalescence distance of 0.2 nm, the shape factor exhibits no significant fluctuation in the temperature range of 300 K–700 K, indicating that the overall cluster shape does not undergo any notable change during this heating process. When the temperature rises to 750 K, the shape factor decreases substantially. For the coalesced clusters with an initial coalescence distance of 0.25 nm, the shape factor first decreases markedly in the range of 300 K–550 K, and then shows a gradual decreasing trend. When the temperature rises from 750 K to 800 K, the shape factor decreases significantly again. When *D_c_* = 0.35 nm, the shape factor of the coalesced clusters first increases slightly and then decreases gradually over the temperature range of 300 K–550 K, indicating that the overall cluster shape is initially elongated and subsequently tends toward a more spherical morphology. For the Cu-Ag clusters coalesced in the edge-to-edge configuration at 300 K, the shape factor is largest for those with an initial coalescence distance of 0.15 nm. The shape factor values for clusters with initial coalescence distances of 0.1, 0.2, 0.25, and 0.3 nm are relatively close to one another, while that for the clusters with an initial coalescence distance of 0.35 nm is considerably lower than the above-mentioned clusters, indicating that the alloy clusters with *D_c_* = 0.35 nm exhibit the highest degree of coalescence at this temperature. During the initial stage of heating, the shape factors of these six clusters exhibit a fluctuating downward trend, indicating that the degree of coalescence of the alloy clusters gradually improves. Upon entering the high-temperature regime, the shape factors show smaller fluctuations and remain roughly in the range of 1.0–1.2, suggesting that the overall configuration of the clusters is approximately spherical. When the two clusters contact in the facet-facet mode with a contact distance of 0.3 nm at 300 K, the shape factor of the cluster is 2.42, which is significantly higher than that of other clusters. This indicates that the Cu and Ag clusters exhibit a low coalescence degree and weak atomic diffusion at this temperature.

**Figure 6:**
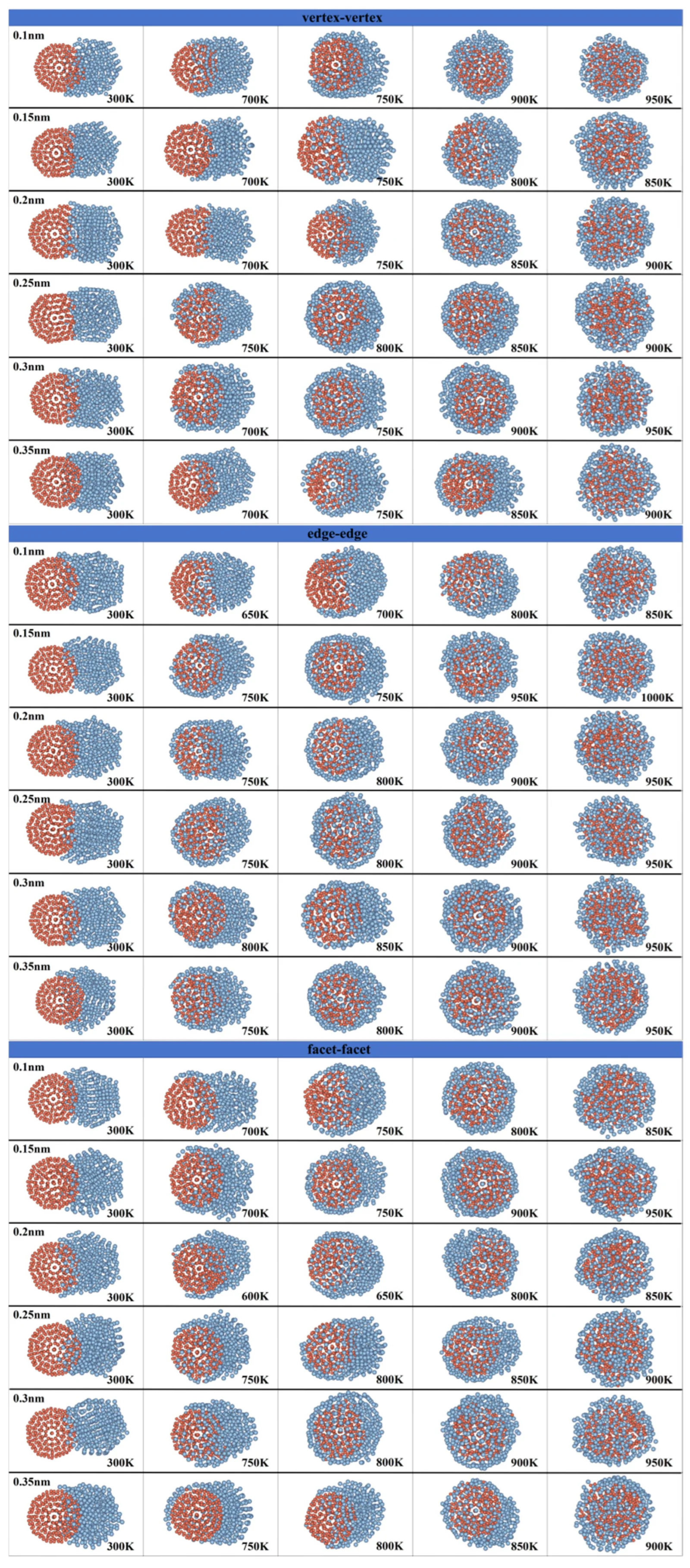
Packing structures of the coalesced clusters during heating.

Figure 6 presents the packing structures of the alloy clusters during heating, where the blue balls denote Ag atoms and the orange balls represent Cu atoms. When the coalescence mode is vertex-to-vertex, at 300 K, the packing configurations of the coalesced clusters at the six different coalescence distances are relatively similar. Specifically, some Ag atoms in the outer shell of the Ag cluster begin to deviate from their initial positions and diffuse along the surface of the Cu cluster, while the Cu cluster retains its Ih packing structure. This behavior is primarily attributed to the fact that coalescence results from rapid surface diffusion of atoms from high-curvature unstable regions toward low-curvature more stable regions [41,42]. When *D_c_* = 0.1 nm and the temperature rises to 700 K, the diffusion of Ag atoms intensifies with increasing temperature, such that the Ag atoms can partially wrap around the Cu cluster, forming a core@partial-shell packing structure. At 900 K, the Ag atoms completely encapsulate the Cu atoms. After atomic rearrangement, the Cu atoms together with the majority of Ag atoms form a new Ih packing structure, while a small number of remaining Ag atoms adopt a disordered arrangement. The core and shell of the bimetallic Cu-Ag cluster are determined by the relative free enthalpy of different atomic arrangements. The configuration with the lowest free enthalpy corresponds to the thermodynamically favored core–shell arrangement, where Ag atoms preferentially segregate onto the outer shell and Cu atoms constitute the inner core. When the temperature further increases to 950 K, the alloy cluster enters a molten state, in which both Cu and Ag atoms are arranged in a disordered manner. For the four clusters with initial coalescence distances of 0.15, 0.2, 0.25, and 0.3 nm, as the temperature increases, they first form a core@partial-shell packing structure and subsequently transform into an incomplete Ih packing structure. When *D_c_* = 0.35 nm, the cluster remains in a core@partial-shell packing structure as the temperature rises to 850 K. At 900 K, the Cu-Ag coalesced cluster completely melts. This also explains why the average potential energy of the cluster in Figure 4a increases slowly without any abrupt change over the temperature range of 300–850 K. When the coalescence mode is edge-to-edge, at 300 K, the cluster with an initial coalescence distance of 0.35 nm exhibits the highest degree of coalescence. In the intermediate-to-high temperature regime, these six clusters predominantly adopt either a core@partial-shell packing structure or an incomplete Ih packing structure. When the coalescence mode is facet-to-facet, for the cluster with an initial coalescence distance of 0.3 nm at 300 K, both the Cu and Ag clusters largely retain their Ih structures, with only a small number of Ag atoms undergoing diffusion. This observation is consistent with the highest shape factor value for this cluster shown in Figure 5c. Prior to melting, although the alloy clusters with initial coalescence distances ranging from 0.15 to 0.35 nm all form icosahedral packing structures, their outer shells exhibit some degree of disorder and aggregation among a portion of the Ag atoms.

## 4. Conclusions

In this work, molecular dynamics simulations have been performed to investigate the coalescence of Ag_309_ and Cu_309_ clusters with initial Ih geometry and the structural evolution of the resulting Cu-Ag alloy clusters during heating, with particular focus on the effects of initial coalescence distance (*D_c_* = 0.1–0.35 nm) and contact orientation (vertex-to-vertex, edge-to-edge, and facet-to-facet). The activation energy for coalescence is found to depend on both the initial coalescence distance and the contact orientation. At the largest coalescence distances, the facet-to-facet orientation exhibits the highest activation energy, followed by the vertex-to-vertex orientation, with the edge-to-edge orientation showing the lowest. During the initial coalescence stage, the free energy remains essentially constant before the clusters come into contact, after which distinct evolutionary behaviors emerge depending on the orientation. In addition, for both vertex-to-vertex and edge-to-edge coalescence, the initial free energy decreases with increasing coalescence distance. At the same coalescence distance, the free energy of vertex-to-vertex coalesced clusters is significantly lower than that of the other two orientations. In the early heating stage, the shape factor of the Cu-Ag clusters exhibits an overall fluctuating downward trend, indicating a progressive improvement in the degree of coalescence. At elevated temperatures, the shape factor remains within the range of 1.0–1.2, suggesting that the alloy clusters adopt a nearly spherical overall configuration. Atomic packing analysis reveals that, at 300 K, in the vertex-to-vertex coalesced clusters, Ag atoms diffuse along the surface of the Cu cluster while the Cu cluster retains its Ih structure. Upon heating, the coalesced clusters successively undergo the formation of a core@partial-shell structure, an incomplete Ih packing, and ultimately complete melting at high temperatures. The six edge-to-edge coalesced clusters predominantly exhibit either core@partial-shell or incomplete Ih packing structures in the intermediate-to-high temperature regime. For the facet-to-facet coalesced clusters with *D_c_* = 0.15–0.35 nm, although Ih packing structures are formed prior to melting, their outer shells exhibit some degree of disorder and aggregation among a portion of the Ag atoms.

## Figures and Tables

**Table 1:** Activation energy *E_A_* of the clusters.

*E_A_*/eV	0.1 nm	0.15 nm	0.2 nm	0.25 nm	0.3 nm	0.35 nm
vertex-vertex	0.00139	0.00373	0.00385	0.00420	0.00416	0.00460
edge-edge	0.00125	0.00228	0.00213	0.00488	0.00151	0.00438
facet-facet	0.00726	0.00471	0.00391	0.00635	0.00485	0.00401

## Data Availability

The data that support the findings of this study are available from the corresponding author upon reasonable request. The data are not publicly available because they will be used for further unpublished analyses.
